# Neuroprotective effects of compounds with antioxidant and anti-inflammatory properties in a *Drosophila *model of Parkinson's disease

**DOI:** 10.1186/1471-2202-10-109

**Published:** 2009-09-01

**Authors:** Katharina Faust, Stephan Gehrke, Yufeng Yang, Lichuan Yang, M Flint Beal, Bingwei Lu

**Affiliations:** 1 Department of Pathology, Stanford University School of Medicine, Stanford, CA 94305, USA; 2 GRECC/VA Palo Alto Health Care System, 3801 Miranda Avenue, Palo Alto, CA 94304, USA; 3 Department of Neurology, Cornell University Medical College, 525 East 68th street, New York, NY 10021, USA

## Abstract

**Background:**

Parkinson's disease (PD) is the most common movement disorder. Extrapyramidal motor symptoms stem from the degeneration of the dopaminergic pathways in patient brain. Current treatments for PD are symptomatic, alleviating disease symptoms without reversing or retarding disease progression. Although the cause of PD remains unknown, several pathogenic factors have been identified, which cause dopaminergic neuron (DN) death in the substantia nigra (SN). These include oxidative stress, mitochondrial dysfunction, inflammation and excitotoxicity. Manipulation of these factors may allow the development of disease-modifying treatment strategies to slow neuronal death. Inhibition of DJ-1A, the *Drosophila *homologue of the familial PD gene DJ-1, leads to oxidative stress, mitochondrial dysfunction, and DN loss, making fly DJ-1A model an excellent *in vivo *system to test for compounds with therapeutic potential.

**Results:**

In the present study, a *Drosophila *DJ-1A model of PD was used to test potential neuroprotective drugs. The drugs applied are the Chinese herb celastrol, the antibiotic minocycline, the bioenergetic amine coenzyme Q10 (coQ10), and the glutamate antagonist 2,3-dihydroxy-6-nitro-7-sulphamoylbenzo[f]-quinoxaline (NBQX). All of these drugs target pathogenic processes implicated in PD, thus constitute mechanism-based treatment strategies. We show that celastrol and minocycline, both having antioxidant and anti-inflammatory properties, confer potent dopaminergic neuroprotection in *Drosophila *DJ-1A model, while coQ10 shows no protective effect. NBQX exerts differential effects on cell survival and brain dopamine content: it protects against DN loss but fails to restore brain dopamine level.

**Conclusion:**

The present study further validates *Drosophila *as a valuable model for preclinical testing of drugs with therapeutic potential for neurodegenerative diseases. The lower cost and amenability to high throughput testing make *Drosophila *PD models effective *in vivo *tools for screening novel therapeutic compounds. If our findings can be further validated in mammalian PD models, they would implicate drugs combining antioxidant and anti-inflammatory properties as strong therapeutic candidates for mechanism-based PD treatment.

## Background

PD is the second most common neurodegenerative disease in the western world and the single most common movement disorder. Over 1 million people in the United States are affected [1]. Symptoms including rigidity, resting tremor, bradykinesia and postural instability are due to degeneration of the brain's nigrostriatal system with progressive loss of DNs in the substantia nigra pars compacta (SNpc), accompanied by depletion of the transmitter dopamine in the striatum. Current pharmacological therapy for PD ameliorates symptoms for a limited period of time, without retarding or reversing disease progression. Currently administered drugs work by increasing the concentration of functional dopamine in the striatum by one of a number of mechanisms: replacing dopamine itself (L-Dopa), inhibiting dopamine-degrading enzymes to prolong its half-life (Entacapone, Selegeline), or mimicking the effect of dopamine on its receptors with dopamine agonists (Bromocriptine, Pergolide, Pramipexole, etc). L-Dopa has remained the single most effective PD drug since its introduction decades ago [2,3].

New treatment strategy aimed at slowing or halting DN death is desired. In the course of elucidating pathogenic events that eventually lead to PD, at least four major mechanisms have been identified: oxidative stress, protein aggregation, inflammation and excitotoxicity [4,5]. It is assumed that these pathways constitute a complex network of events that eventually leads to DN death. Consequently, effective disease-modifying therapy would require addressing a combination of neurodegenerative mechanisms within the SN.

Even though the vast majority of PD cases are sporadic idiopathic forms, recent identification of a number of genes (PARK 1-11) responsible for rare familial cases has provided tremendous insight into the pathogenesis of the disease. The rationale behind studying rare genetic forms of a common sporadic disease is the assumption that they share key biochemical pathways. Of the ten genetic loci linked to familial PD, six gene products have been characterized so far: α-Synuclein, Parkin, UCH-L1, DJ-1, PINK1, and LRRK2 [5,6]. DJ-1 is a relatively small, evolutionarily conserved protein belonging to the ThiJ/PfpI/DJ-1 family. Members of the ThiJ/PfpI/DJ-1 family include chaperones, proteases and transcriptional regulators [7], yet DJ-1's biochemical function relevant to PD remains to be defined. DJ-1 has been implicated in diverse cellular processes, including cellular transformation and tumorigenesis [8,9], transcriptional regulation and RNA binding [10], androgen receptor signaling [11,12], spermatogenesis [13], and oxidative stress response [14,15]. *In vitro *studies showed that DJ-1 responds to oxidative stress induced by paraquat exposure, with a shift of its iso-electric point towards a more acidic form (from pI 6.2 to pI 5.8) [15]. Postmortem analysis of PD brains detected higher concentrations of the acidic DJ-1 isoforms, as compared to healthy controls [7]. DJ-1 is a hydrogen peroxide (H_2_O_2_)-responsive protein. H_2_O_2 _exposure oxidizes all its cysteine residues (Cys 46, 53, 106) to cysteine sulfonic acid [16], with Cys 106 being most sensitive. These studies demonstrate a direct modification of DJ-1 protein by reactive oxygen species (ROS), nourishing the notion that DJ-1 might act as a free radical scavenger or sensor. Cell culture studies of DJ-1 deficient neuronal cells revealed increased susceptibility to H_2_O_2_, MPP^+^, 6-hydroxydopamine, and rotenone [12,17,18], whereas DJ-1 overexpression dramatically reduced H_2_O_2_-induced neuronal death [17,18]. Oxidative stress results in mitochondrial relocalization of DJ-1, which is mediated by oxidation of Cys 106 [19]. Consistent with DJ-1 playing an important role in oxidative stress response, genetic studies in *Drosophila *and mice showed accumulation of ROS and increased sensitivity to oxidative stimuli, including H_2_O_2 _and MPTP, in *DJ-1 *mutants [18,20,22].

Minocycline is a member of the tetracycline group of antibiotics. Minocycline has been found to have additional anti-inflammatory and antioxidant properties independent of its antibacterial activity [23,26], which may be useful for the treatment of neurodegenerative diseases including PD. Minocycline has also been shown to have neuroprotective effects in animal models of other pathological conditions, including ischemia and stroke, traumatic brain injury, amyotrophic lateral sclerosis (ALS), multiple sclerosis (MS), Huntington's disease (HD), and the MPTP model of PD in mouse [27,30]. Potency appears to arise from the modulation of inflammatory cytokine release, microglia activation, nitric oxide production, matrix metalloprotease activation, and apoptotic cell death. Minocycline has an oral bioavailability of almost 100% and its absorption, unlike other tetracyclines, is not reduced by ingestion with food [31]. High lipophilicity allows its easy diffusion into brain tissues. Minocycline also has a lower urinary excretion than other tetracyclines and is thus safer in elderly patients with impaired renal function [32].

Celastrol is a triterpene extracted from the root bark of an ivy-like, creeping plant called Triperygium wilfordii (TW) that is indigenous to Southern China. Extracts of the plant have had a long history of use in traditional Chinese medicine for treating fever, chills, edema and joint pain, conditions commonly associated with inflammation [33]. Celastrol was found to suppress microglial cell activation, release of the inflammatory cytokines TNF-α and IL-1β by human macrophages and monocytes, and the production of nitric oxide (NO) by iNOS [33]. Furthermore, celastrol was demonstrated to be a potent inhibitor of induced lipid peroxidation in rat liver mitochondria, exhibiting over 15-fold more antioxidant potency than α-tocopherol [34]. Celastrol protected both the outer and inner mitochondrial membrane from peroxidation, possibly mediated by its radical-scavenging dienonephenol moiety, while the anionic carboxyl group protects the inner membrane from radical attacks by stabilizing its negative surface charge. In a rat model of Alzheimer's disease (AD), celastrol improved memory and learning in psychomotor-activity tests (PMA) [33]. Low nanomolar concentrations of the drug showed efficacy in all the studies mentioned above.

CoQ10 (also known as ubiquinone) is composed of a quinone ring and a 10-isoprene unit tail. It is an obligatory cofactor in the mitochondrial respiratory chain. As a bioenergetic agent, it serves as an electron acceptor in complexes I and II/III of the electron transport chain. Mitochondrial dysfunction has been frequently observed in PD, and several lines of evidence support its causative role in disease pathogenesis. For example, a 30% to 40% reduction in complex I activity was observed in sporadic PD, and MPTP induces parkinsonism by inhibiting complex I activity [35]. CoQ10 is also a potent antioxidant distributed in all membranes throughout the cell. It is able to work in concert with α-tocopherol and participates in the recovery of cells from oxidative stress [36,37]. Levels of coQ10 measured in mitochondria from PD patients were significantly lower than in age-matched controls [38], while at the same time the percentage of oxidized coQ10 was relatively increased [39]. In *in vitro *models, coQ10 could protect against MPP^+ ^and rotenone induced toxicity [40,42]. In animal models of ALS and HD, coQ10 treatment also showed beneficial effects [43,44]. In the MPTP mouse model of PD, oral treatment of young mice with coQ10 and nicotiamide attenuated the effect of low dose MPTP administration, while coQ10 alone attenuated dopamine depletion in the striatum [45]. CoQ10 treatment (200 mg/kg/day) in aged (1 year old) MPTP-treated mice, whose nervous system might already exhibit degenerative changes, showed that it significantly attenuated MPTP-induced loss of striatal dopamine and loss of TH-immunoreactive fibers in the striatum [46]. CoQ10 administration can increase mitochondrial content of coQ10 in the cortex of 1-year-old rats [43]. Similar promising results have subsequently been generated using a monkey MPTP model of PD [47]. CoQ10 is extremely lipophilic, making it easy to cross the BBB. Its absorption is improved by the inclusion of lipid in the formulation and by taking it with food.

NBQX (2,3-dihydroxy-6-nitro-7-sulphamoylbenzo[f]-quinoxaline) is a potent competitive AMPA-receptor antagonist, belonging to the quinoxalinediones group [48]. AMPA-selective glutamate receptor antagonists constitute potential neuroprotective agents by counteracting the excitotoxic effects of excess glutamate. In the MPP^+ ^mouse model of PD, AMPA receptor antagonists were shown to have greater therapeutic potential than NMDA receptor antagonists [49]. The quinoxalindione derivatives were discovered in 1988 and are still undergoing intensive study [50]. NBQX exhibits improved AMPA receptor selectivity compared to earlier quinoxalindiones [51]. It has systemic activity and was first shown to have therapeutic effects by protecting against cerebral ischemia after carotid artery occlusion in mice [51]. NBQX has thus been used as the antagonist of choice in many "*in vivo*" and "*in vitro*" models. Besides its anti-stroke properties, NBQX also showed efficacy against PD [52], demyelinating disorders [53], and trauma [54].

Given that celastrol, minocycline, coQ10, and NBQX have different mechanisms of action and all shown efficacy in various neurological disease models, we decided to test their efficacy in a *Drosophila *DJ-1 model of PD. There are two DJ-1 homologues in *Drosophila*, DJ-1A and DJ-1B. Despite the fact that DJ-1A is more closely related to human DJ-1 at the sequence level, DJ-1B has been the main focus of research simply because of its ubiquitous and higher level of expression. It should be pointed out that DJ-1A, despite its low level of expression, is expressed in adult brain and is inducible under certain conditions [22]. Results on DJ-1B function in stress response and DN survival using various genetic mutants have been divergent [21,22,55]. Studies on DJ-1A function in stress response and DN survival using two different DJ-1A RNAi lines by independent groups have been consistent [20,56], although one study reported that a genomic deletion mutant of DJ-1A, in which the entire DJ-1A locus and some surrounding sequence were deleted, showed stress sensitivity but no DN loss [21]. The lack of DN loss phenotype in the DJ-1A genomic deletion mutant could be caused by a genome-wide compensatory response to the deletion of DJ-1A; alternatively, the phenotypes induced by tissue-specific DJ-1A RNAi may require some non cell-autonomous function of DJ-1A. The efficiency of the DJ-1A RNAi transgene used in this study and evidence that the DJ-1A RNAi phenotype is unlikely due to "off target" effects have been presented in previous studies [20]. Given that the DJ-1A RNAi model recapitulates two features of PD: loss of DN and reduction of brain dopamine levels [20,56], we decided to test the drugs on this model. We expect that results from this study will help understand the role of DJ-1 dysfunction in PD pathogenesis and validate the usefulness of fly PD models in pharmacological studies.

## Results

**Figure 1:**
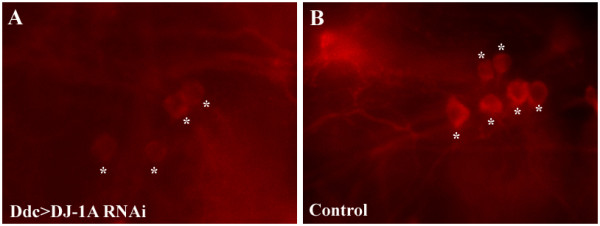
**Whole-mount confocal immunofluorescence analysis of TH+ neurons in the DMC of *Ddc-Gal4>DJ-1A RNAi *flies**. Whole-mount brain samples of 25-day old *Ddc-Gal4>UAS-DJ-1A RNAi *(**A**) or control *Ddc-Gal4>UAS-GFP *(**B**) flies were immunostained with anti-TH antibody and TH-positive immunofluorescence signals were detected by confocal microscopy. Representative confocal images of the TH+ neurons in the DMC are shown. Asterisks mark the identifiable TH+ neurons. Note that not only was there a reduction of TH+ neurons in *Ddc-Gal4>UAS-DJ-1A RNAi *flies compared to the control, the intensity of TH immunoreactivity was also reduced in the remaining neurons.

To evaluate the effects of potential neuroprotective drugs *in vivo*, DN survival and dopamine content of the brain were measured in a fly *DJ-1A *RNAi model of PD [20]. The effects of the drugs celastrol, minocycline, NBQX, and coQ10 on DJ-1A RNAi-induced dopaminergic dysfunction and degeneration were investigated. One pathological hallmark of PD is a progressive loss of DNs in the SN. It has been shown that DNs in a circumscribed brain region, the dorsomedial cluster (DMC), degenerate in *Drosophila *models of PD [57,58]. Thus, serial frontal sections across the brains of aged flies were first immunostained using the anti-tyrosine hydroxylase (TH) antibody that specifically detects dopamine-synthesizing neurons. Immunopositive neurons of the DMC were detected with the peroxidase/DAB staining methods and quantified. Although the validity of the paraffin section peroxidase/DAB staining method originally used to detect DN loss in the fly α-Syn model was questioned [57,59,62], later studies using confocal analysis of whole-mount TH immunostaining confirmed α-Syn toxicity in fly DNs [63]. These results suggest that both the paraffin section peroxidase staining and whole-mount immunostaining methods are valid for detecting TH+ neurons. Consistently, we could observe the DN loss phenotype in DJ-1A RNAi flies using either the whole-mount confocal microscopy (Fig. 1), or the paraffin section peroxidase staining method [20]. In DJ-1A RNAi flies, the age-dependent loss of DNs was shown to be preceded and accompanied by a decrease of dopamine levels in the brain [20]. Therefore, in the second set of experiments we studied whether the drugs would also affect levels of brain dopamine, as measured by HPLC analysis of fly head homogenates.

### Age-dependent loss of DNs and reduction of brain dopamine level in Ddc-Gal4>DJ-1A RNAi flies

**Figure 2:**
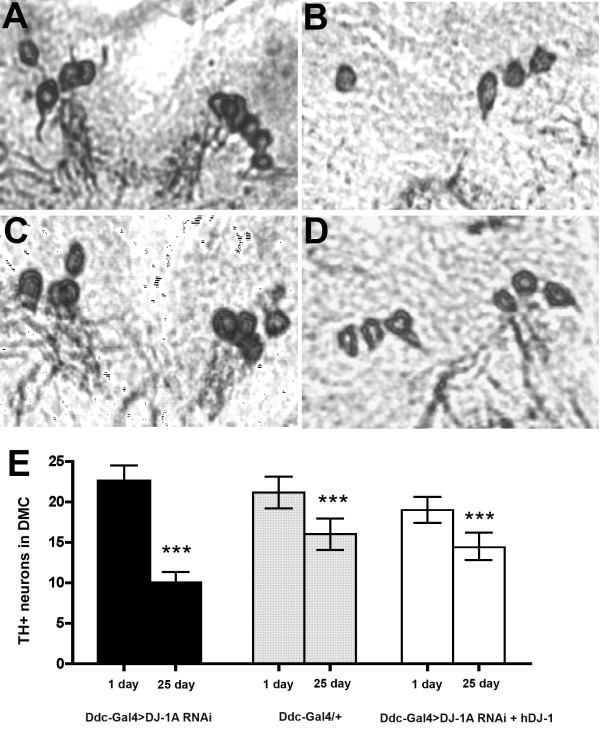
**Immunohistochemical analysis showing age-dependent loss of TH+ neurons in the DMC of *Ddc-Gal4>DJ-1A RNAi *flies**. **A-D**: Frontal paraffin sections of adult fly brain were reacted with anti-TH antibody and detected with the peroxidase/DAB staining method. Representative sections containing DA neurons in the DMC were shown for each genotype. **A, B**: *Ddc-Gal4>DJ-1A RNAi *flies; C, D: *Ddc-Gal4/+ *flies. **A, C**: TH-immunostaining of 1-day-old flies; **B, D**: TH-immunostaining of 25-day-old flies. **E**: Statistical analysis of the number of DNs within the DMC of *Ddc-Gal4/+*, *Ddc-Gal4>DJ-1A RNAi*, and *Ddc-Gal4>DJ-1A RNAi+hDJ-1 *genotyped flies. Flies were fed with wet yeast paste. Between 8-25 fly heads for each genotype and age were evaluated. Columns depict mean with standard deviation of the mean (SD). ***: *P *< 0.0001 in ANOVA with Dunnett's post test.

An age-dependent loss of DNs in the DJ-1A RNAi flies was observed in two independent studies where the dopaminergic specific transcriptional regulator *TH-GAL4 *or *dopa decarboxylase (Ddc)*-Gal4 were used to direct the expression of two different DJ-1A RNAi transgenes [20,56]. Since the culture conditions used in those previous studies were different from the current study, in which animals were fed with yeast paste or drug-containing yeast paste, *Ddc-Gal4>DJ-1A RNAi *flies cultured under yeast paste condition were first analyzed for DN number and brain dopamine content. Young, 1-day-old *Ddc-Gal4>DJ-1A RNAi *flies and age-matched *Ddc-Gal4/+ *control flies showed no significant difference in their number of TH-positive (TH+) neurons within the DMC (22.6 ± 1.98 in DJ-1A RNAi flies vs. 21.20 ± 1.97 in controls, *P *= 0.98) (Fig. 2). With age, both DJ-1A RNAi flies and control flies showed a reduction of TH+ neurons (Fig. 2). The age-dependent loss of TH+ neurons was more substantial when DJ-1A was deficient: DJ-1A RNAi flies aged for 25 days suffered a 55.3% decrease of TH+ neurons within the DMC, as compared to 1-day-old flies of the same genotype (22.6 ± 1.98 at 1 day vs. 10.1 ± 1.31 at 25 days), whereas 25-day-old control flies only exhibited a 24.3% loss compared to 1-day-old flies of the same genotype (21.2 ± 1.97 at 1 day vs. 16.1 ± 1.95 at 25 days). Thus, at 25 days of age, the number of TH+ neurons within the DMC of *Ddc-Gal4>DJ-1A RNAi *flies was 62.8% of age-matched *Ddc-Gal4/+ *controls (*P *< 0.0001 in ANOVA with Dunnett's post test). Another independent transgenic fly line for the same *UAS-DJ-1A RNAi *transgene showed similar degree of TH+ neuron loss when driven by *Ddc-Gal4 *driver (data not shown). Consistent with the DJ-1A RNAi effect being caused by inhibition of DJ-1A function instead of some "off-target" effect, co-expression of human DJ-1 (hDJ-1) was able to rescue DJ-1A RNAi-induced loss of TH+ neurons (Fig. 2). In previous studies, control flies (*TH-Gal4/+*) aged at 35 days did not show obvious DN number change [20]. The difference between the control flies used in previous studies and the current study is most likely due to the different feeding conditions (yeast paste vs. standard fly food), since the *TH-Gal4/+ *control flies used in the previous study also showed a decrease of TH+ neurons at 25 days of age when grown in yeast-fed condition (data not shown).

We also measured brain dopamine contents in *Ddc-Gal4>DJ-1A RNAi *and *Ddc-Gal4/+ *control flies fed with yeast paste, using HPLC analysis of fly head extracts. Brain dopamine levels in adult flies show dramatic age-dependent reduction [20], such that at 25 days of age, a large number of fly heads would be required to ensure quantitative and reliable measurement of brain dopamine levels. We therefore measured younger flies when dopamine level can be reliably measured. Even when assayed at 10 days of age, *Ddc-Gal4>DJ-1A RNAi *flies already showed more than 50% reduction of brain dopamine level compared to *Ddc-Gal4/+ *control flies (Fig. 4). At this age, no obvious change in DN number was observed (data not shown), suggesting that brain dopamine level change occurred before DN loss instead of being caused by that. These results showed that DJ-1A RNAi-induced DN degeneration and reduction of brain dopamine content can be observed under both standard feeding conditions previously reported [20] and yeast feeding condition reported here.

### The effects of celastrol on DN number and brain dopamine content

**Figure 3:**
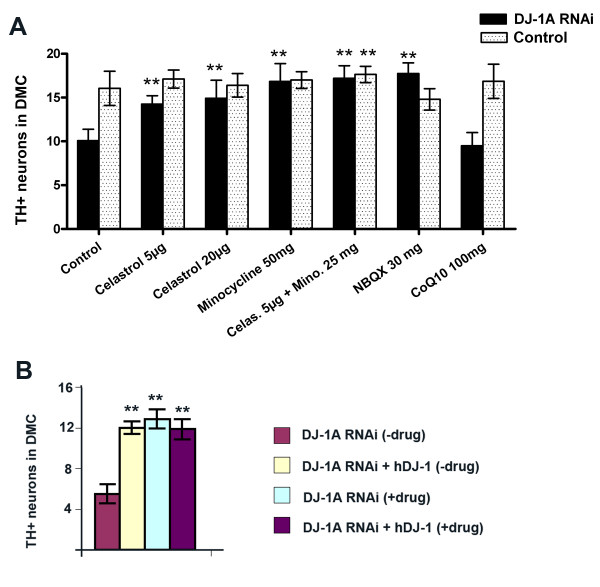
**Statistical analysis of the effects of the various drugs on DN number in *Ddc-Gal4>DJ-1A RNAi *flies**. **A**: The numbers of DNs in the DMC of drug-treated 25-day-old *Ddc-Gal4>DJ-1A RNAi *flies was determined by TH-immunostaining of paraffin brain sections. These were compared with untreated *Ddc-Gal4>DJ-1A RNAi *flies or control *Ddc-Gal4/+ *flies with or without drug treatment. Animals were raised at 25°C. **B**: The numbers of DNs in the DMC of 25-day-old *Ddc-Gal4>DJ-1A RNAi *and *Ddc-Gal4>DJ-1A RNAi+hDJ-1 *flies with or without celastrol treatment (20 μg/ml) were determined by whole-mount TH-immunostaining. Animals were raised at 29°C. Note that at 29°C the DJ-1A RNAi effect is stronger than 25°C. Results are the mean ± SD of 8-25 sectioned heads for each genotype and drug treatment. **: *P *< 0.01 in ANOVA with Dunnett's post test.

**Figure 4:**
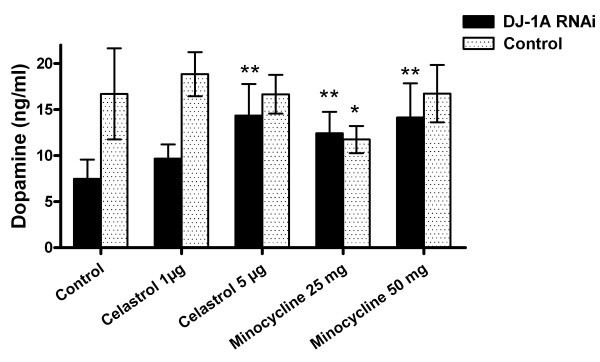
**HPLC analysis of the effects of celastrol and minocycline treatments on brain dopamine content**. Head homogenates of 10-day-old *Ddc-Gal4>DJ-1A RNAi *flies treated with celastrol or minocycline were measured for dopamine content by HPLC. These were compared with untreated *Ddc-Gal4>DJ-1A RNAi *flies and control *Ddc-Gal4/+ *flies with or without drug treatments. Results are the mean ± SD of 21 homogenized heads for each genotype and drug treatment. *: *P *< 0.05 and **: *P *< 0.01 in ANOVA with Dunnett's post test.

We next tested the effects of drug candidates in modulating DJ-1A RNAi-induced dopaminergic phenotypes. We found that celastrol treatment both prevented DN loss and protected against dopamine depletion observed in *Ddc-Gal4>DJ-1A RNAi *flies. The effects of celastrol treatments are shown in Fig. 3 and Fig. 4. Immunostaining of TH+ neurons in the DMC of *Ddc-Gal4>DJ-1A RNAi *flies treated with celastrol at 5 μg/ml revealed a significant increase in the number of these neurons (10.1 ± 1.3 in untreated flies vs. 14.3 ± 1.0 in treated flies, a mean increase of 41%, *P *< 0.01). When treated with a higher dose of celastrol (20 μg/ml), a mean increase of 48% was observed. As a control, we found that *Ddc-Gal4>DJ-1A RNAi+hDJ-1 *flies with or without celastrol treatment had similar number of TH+ neurons (Fig. 3B).

To further ascertain the neuroprotective effects of celastrol, brain dopamine levels were measured in 10-day-old flies treated with celastrol. Our initial dosing experiments showed that lower doses of celastrol were sufficient to preserve brain dopamine level. Therefore we used two different doses in the lower range, 1 μg/ml and 5 μg/ml, in brain dopamine measurement experiments (Fig. 4). After feeding with 1 μg/ml celastrol, mean dopamine levels in *Ddc-Gal4>DJ-1A RNAi *flies were increased by 30% (9.7 ± 1.5 ng/ml in treated vs. 7.5 ± 2.1 ng/ml in untreated animals). However, this increase showed no statistical significance (*P *> 0.05). Treatment of flies with a higher dose of celastrol (5 μg/ml) significantly increased dopamine levels to 14.4 ± 3.4 ng/ml, a 92% increase (*P *< 0.01). Treatment of *Ddc-Gal4>DJ-1A RNAi *flies with 1 μg/ml or 5 μg/ml celastrol therefore preserved dopamine content at 58% or 86% of wild type level, respectively.

The protective effects of celastrol appeared to be selective to the *Ddc-Gal4>DJ-1A RNAi *flies, since celastrol treatment in control *Ddc-Gal4/+ *flies did not produce any significant changes in either TH+ neuron number or brain dopamine level. TH+ neuron numbers in *Ddc-Gal4/+ *flies were 17.1 ± 1.0 in 5 μg/ml celastrol-fed vs. 16.1 ± 2.0 in yeast-fed only conditions (*P *> 0.05), and 16.4 ± 1.4 in 20 μg/ml celastrol-fed vs. 16.1 ± 2.0 in yeast-fed only conditions (*P *> 0.05). Brain dopamine levels in *Ddc-Gal4/+*flies were 18.8 ± 2.4 ng/ml in 1 μg/ml celastrol-fed and 16.7 ± 2.1 ng/ml in 5 μg/ml celastrol-fed conditions, compared to 16.7 ± 5.0 ng/ml in yeast-fed only condition. These changes were not statistically significant (*P *> 0.05 in both comparisons).

### The effect of minocycline on DN number and brain dopamine content

Treatment of *Ddc-Gal4>DJ-1A RNAi *flies with minocycline also resulted in attenuation of both DN loss and dopamine depletion in the brain (Fig. 3 and Fig. 4). DJ-1A RNAi flies that received daily feeds of minocycline-containing yeast paste (50 mg/ml) for 25 days showed a significant increase of TH+ neurons within the DMC compared to flies of the same genotype fed with yeast only (Fig. 3). The mean number of TH+ neurons in the DMC of minocycline (50 mg/ml) treated DJ-1A RNAi flies was 16.8 ± 2.0 (mean ± SD), as compared to 10.1 ± 1.3 in DJ-1A RNAi flies fed with yeast only. Thus, treatment with 50 mg/ml minocycline resulted in a recovery of TH+ DMC neurons in *Ddc-Gal4>DJ-1A RNAi *flies to wild type levels (16.1 ± 2.0 at 25 days), which represented a 67% increase compared to the same genotyped flies without drug treatment.

We further examine the effects of minocycline on dopamine concentrations in head homogenates of 10-day-old *Ddc-Gal4>DJ-1A RNAi *flies. Daily oral uptake of minocycline attenuated dopamine depletion in a dose-dependent manner (Fig. 4). *Ddc-Gal4>DJ-1A RNAi *flies treated with 25 mg/ml or 50 mg/ml minocycline showed mean dopamine concentrations of 12.4 ± 2.3 ng/ml (*P *< 0.01) or 14.1 ± 3.7 ng/ml (*P *< 0.01), respectively, while the same genotyped flies fed with yeast only had dopamine level of 7.5 ± 2.1 ng/ml. Age-matched *Ddc-Gal4/+ *control flies under yeast-fed condition had dopamine levels at 16.7 ± 5.0 ng/ml. Hence, as compared to the yeast-fed only condition, high dose minocycline almost doubled dopamine levels in *Ddc-Gal4>DJ-1A RNAi *flies, reaching 85% of wild type levels. Feeding with lower dose minocycline (25 mg/ml) also increased dopamine levels, although to a lesser extent, reaching 71% of wild type levels.

Minocycline had no effect on control *Ddc-Gal4/+ *flies, when fed in high doses (50 mg/ml). Neither TH+ neuron number (17.0 ± 1.0 in minocycline-fed condition vs. 16.1 ± 2.0 in yeast-fed condition, *P *> 0.05), nor mean brain dopamine level (16.7 ± 3.1 ng/ml in minocycline-fed condition vs. 16.7 ± 5.0 ng/ml in yeast-fed condition, *P *> 0.05) was significantly different for *Ddc-Gal4/+*flies with or without minocycline treatment. In lower dose minocycline-feeding condition (25 mg/ml), however, a modest reduction of dopamine level was observed in *Ddc-Gal4/+*control flies (11.7 ± 1.5 ng/ml in minocycline-feeding condition vs. 16.7 ± 5.0 ng/ml in yeast-feeding condition, *P *< 0.05), although no significant difference in DN number was observed (data not shown). The significance of this lower-dose minocycline effect on dopamine level is not clear.

### The effects of NBQX on DN number and brain dopamine content

The effect of NBQX treatment on DN number or brain dopamine content was also analyzed. The number of TH+ neurons in the DMC of *Ddc-Gal4>DJ-1A RNAi *flies treated with NBQX (30 mg/ml) was on average 76% higher than age-matched untreated flies of the same genotype (17.7 ± 1.2 in NBQX-fed vs. 10.1 ± 1.3 in yeast-fed conditions, *P *< 0.01). Thus NBQX treatment was sufficient to fully restore DN number to wild type levels at 25 days of age (Fig. 3 and Fig. 6E).

**Figure 5:**
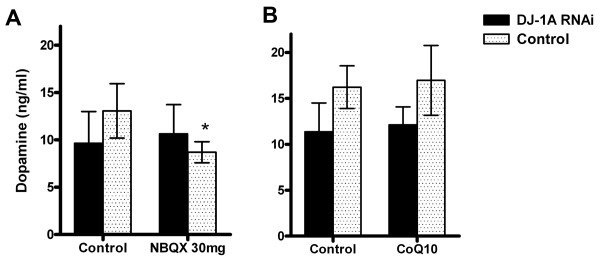
**HPLC analysis of the effects of NBQX and coQ10 treatments on brain dopamine concentration**. Head homogenates of 10-day-old *Ddc-Gal4>DJ-1A RNAi *flies treated with NBQX or coQ10 were measured for dopamine content by HPLC. These were compared with untreated *Ddc-Gal4>DJ-1A RNAi *flies. Control *Ddc-Gal4/+ *flies with or without drug treatment were similarly analyzed. Results are represented as mean ± SD of 21 homogenized heads for each genotype and drug treatment. *: *P *< 0.05 in ANOVA with Dunnett's post test. Note that there appears to be a significant variation in dopamine level between untreated control and DJ-1 RNAi flies used in this experiment and those used in Fig. 4. This could be due to slight variations in animal age, which can have a strong effect on brain dopamine level [20], or due to sensitivity of dopamine stability to variations in each experimental condition.

**Figure 6:**
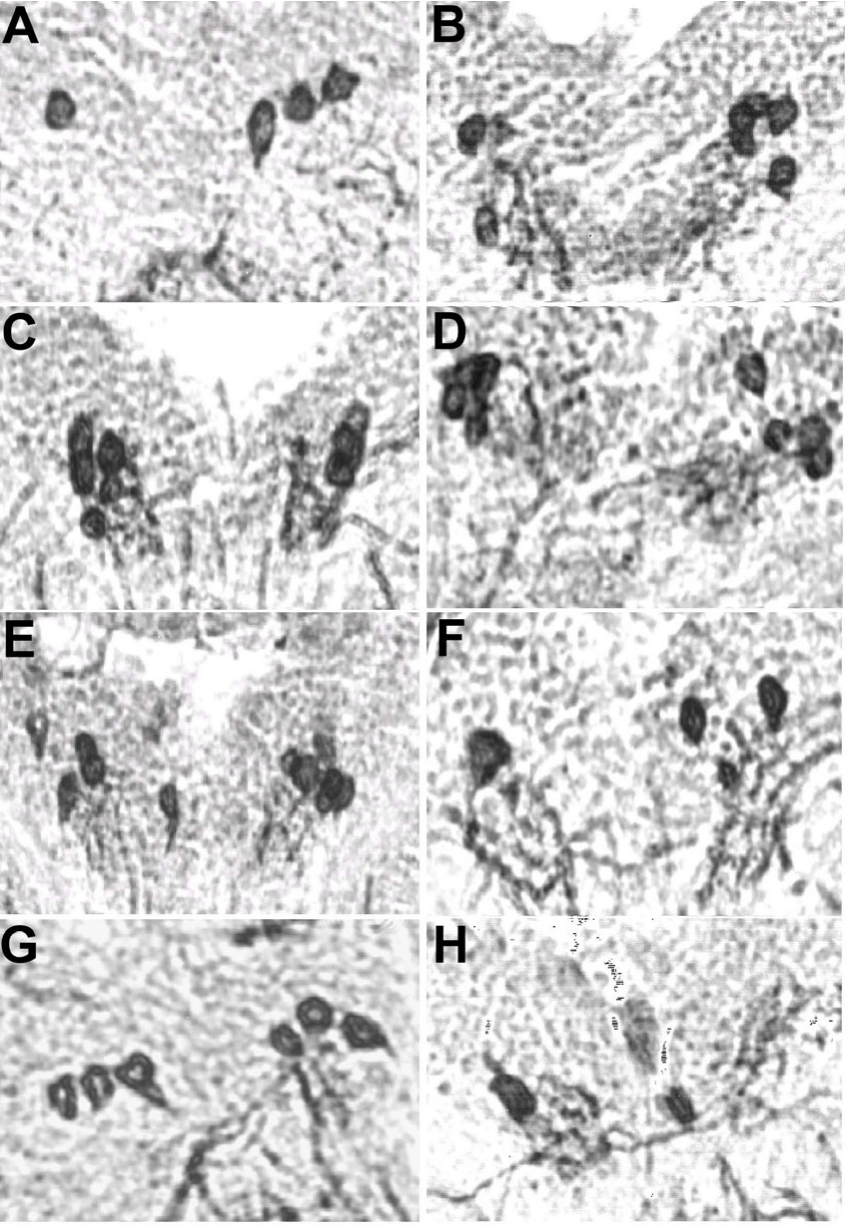
**Immunohistochemical analysis of TH+ DA neurons in the DMC to show the effects of various drug treatments**. Frontal paraffin sections of adult fly brain were stained with anti-TH antibody. Representative sections containing DA neurons in the DMC were shown for each genotype. 25-day-old female flies were analyzed. **A-F**: *Ddc-Gal4>DJ-1A RNAi *flies. **A**: yeast fed no-drug control, **B**: 20 μg/ml celastrol-treated, **C**: 50 mg/ml minocycline-treated, **D**: 5 μg/ml celastrol + 25 mg/ml minocycline-treated, **E**: 30 mg/ml NBQX-treated, **F**: 100 mg/ml coQ10-treated. **G, H**: *Ddc-Gal4/+ *flies. **G**: yeast fed no-drug control, **H**: 30 mg/ml NBQX-treated.

Surprisingly, NBQX-induced DN increase measured by immunohistochemistry was not accompanied by restoration of brain dopamine level assayed by neurochemistry analysis (Fig. 5A). HPLC measurement of brain dopamine concentration showed only a slight increase in *Ddc-Gal4>DJ-1A RNAi *flies treated with NBQX (10.6 ± 3.1 ng/ml in treated animals vs. 9.6 ± 3.4 ng/ml in untreated animals). Thus, in contrast to minocycline and celastrol, NBQX attenuated TH+ neuron loss but failed to prevent dopamine depletion in *Ddc-Gal4>DJ-1A RNAi *flies. Furthermore, in contrast to the effect in *Ddc-Gal4>DJ-1A RNAi *flies, NBQX treatment significantly reduced brain dopamine level in control *Ddc-Gal4/+ *flies (Fig. 5, 13.1 ± 2.9 ng/ml in untreated vs. 8.7 ± 1.1 ng/ml in treated animals, a decrease of 33%; *P *< 0.05). In addition, a trend of decrease (though statistically non-significant) of TH+ neuron number was observed in NBQX-fed *Ddc-Gal4/+ *control flies (16.1 ± 2.0 in untreated vs. 14.8 ± 1.2 in treated animals, *P *> 0.05; Fig. 3 and Fig. 6H), suggesting that at the concentration used (30 mg/ml), NBQX exerted certain toxic effects on the *Ddc-Gal4/+ *control flies.

### The effects of coQ10 on DN number and brain dopamine content

The effects of coQ10 (100 mg/ml) feeding on DN number and dopamine levels in *Ddc-Gal4>DJ-1A RNAi *flies or control *Ddc-Gal4/+ *flies were next analyzed. Mean number of TH+ neurons in the DMC of *Ddc-Gal4>DJ-1A RNAi *flies treated with 100 mg/ml coQ10 was 9.5 ± 1.5, compared with 10.1 ± 1.3 in the untreated condition (*P *> 0.05). *Ddc-Gal4/+ *control flies treated with 100 mg/ml coQ10 contained 16.9 ± 2.0 TH+ neurons in the DMC, compared with 16.1 ± 2.0 in the untreated condition (*P *> 0.05) (Fig. 3, Fig. 6F). As shown in Fig. 5B, mean brain dopamine levels of *Ddc-Gal4>DJ-1A RNAi *flies were 12.1 ± 1.9 ng/ml in 100 mg/ml coQ10-treated vs. 11.4 ± 3.1 ng/ml in the untreated conditions (*P *> 0.05). In *Ddc-Gal4/+ *control flies, brain dopamine levels were 17.0 ± 3.8 ng/ml in 100 mg/ml coQ10-treated vs. 16.2 ± 2.3 ng/ml in the untreated condition (*P *> 0.05) (Fig. 5B). Thus, coQ10 treatments had no significant effect on DA neuron number or dopamine levels in *Ddc-Gal4>DJ-1A RNAi *flies or control *Ddc-Gal4/+ *flies.

### The effects of celastrol on locomotor activity and oxidative stress response

**Figure 7:**
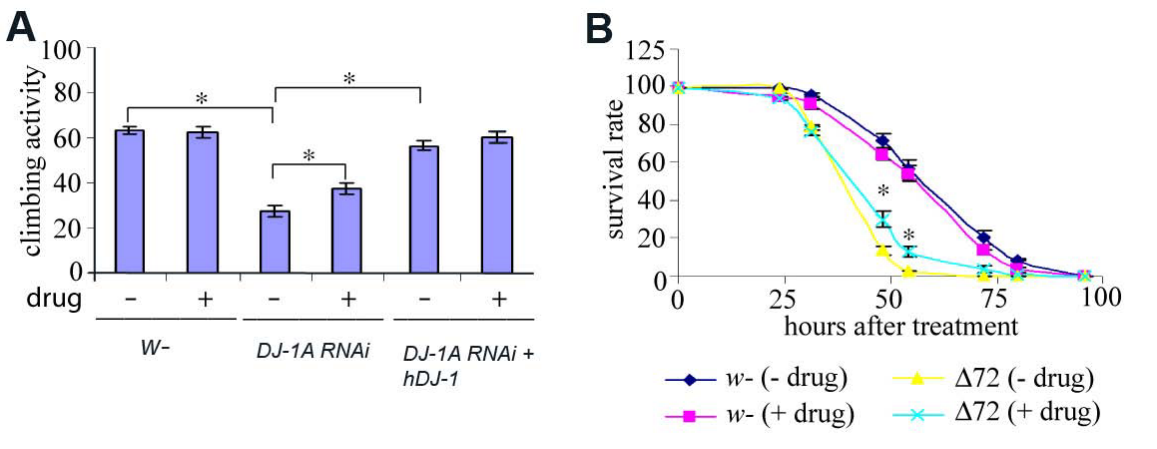
**Effects of celastrol treatment on the climbing activity of DJ-1A RNAi flies and oxidative stress sensitivity of *DJ-1A *genomic deletion flies**. **A**: *Ddc-Gal4>UAS-DJ-1A RNAi *flies, *Ddc-Gal4>UAS-DJ-1A RNAi + hDJ-1 *flies, and control wild type flies were treated with 20 μg/ml celastrol or mock-treated for 20 days before they were subjected to the climbing activity assay, in which the flies were bumped to the bottom of a plastic vial and the percentage of flies climbing to the top half of the vial after 7 seconds was quantified. Asterisks indicate *P *< 0.05 in Student's *t*-test. **B**: *DJ-1A *genomic deletion flies (Δ*72*) and control wild type (*w*^-^) flies were treated with 20 μg/ml celastrol or mock-treated for 20 days before they were subjected to the oxidative stress assay, in which the flies were exposed to 0.5% H_2_O_2 _and the survival rate over a 100 hour period was quantified. Asterisks indicate *P *< 0.05 in Student's *t*-test.

Locomotor dysfunction is frequently observed in *Drosophila *PD models [55,57,64,65]. We found that aged DJ-1A RNAi flies also exhibited reduced climbing activity when compared to control flies. Further, this defect in climbing activity was rescued by co-expressing hDJ-1 (Fig. 7A). To test whether celastrol treatment may modify this phenotype, we fed newly eclosed flies with drug-containing yeast-paste. After 20 days of drug treatment, their climbing activity was assayed. While celastrol treatment did not significantly affect the climbing activity of wild type control flies or DJ-1A RNAi flies rescued by hDJ-1, it significantly improved the climbing activity of the DJ-1A RNAi flies (Fig. 7A), even though the activity was not fully restored to wild type level.

We also tested the effects of celastrol in a genomic deletion mutant of DJ-1A, *DJ-1A(Δ72)*. In this mutant, the entire DJ-1A locus and some surrounding sequence were deleted [21]. Both wild type control flies and *DJ-1A(Δ72) *flies were raised under either celastrol-fed or yeast-fed conditions. After 20 days of treatment, the animals were subjected to H_2_O_2 _treatment. We found that while celastrol treatment did not affect the survival rate of wild type control flies under H_2_O_2_-induced oxidative stress, it conferred moderate but statistically significant improvement on the survival of *DJ-1A(Δ72) *flies (Fig. 7B). Together, these results support that celastrol treatment could ameliorate various cellular deficits associated with DJ-1A inactivation.

## Discussion

Loss-of-function mutations in the DJ-1 gene have been associated with inherited forms of PD. Although familial forms of PD are rare, studies of their gene products can provide valuable insights into the pathogenic mechanisms underlying idiopathic PD. The isolation of familial PD genes has helped to create animal models of the disease, which can be used for pharmacological testing. In the present study, the neuroprotective effects of four drugs, celastrol, minocycline, coQ10 and NBQX, were studied in a *Drosophila *DJ-1 model of PD. The parameters used here, brain dopamine level and the survival of DNs within the DMC, are relevant to the pathological hallmarks seen in PD patients. The results shown here therefore have implications for the introduction of new neuroprotective drugs into clinical practice as well as for further understanding the role of DJ-1 in normal dopaminergic physiology and PD pathogenesis.

### Compounds combining antioxidant and anti-inflammatory properties provide neuroprotection in a fly DJ-1A model of PD: celastrol and minocycline studies

As described earlier, neuronal degeneration in PD is accompanied by oxidative damage and inflammation. Oxidative damage, one likely source of which being the metabolites of dopamine, manifests itself through mitochondrial dysfunction and elevated levels of metals and neuromelanin [66]. Inflammation in PD is presumably mediated by activated microglia and their release of inflammatory cytokines [67,70], as well as nitric oxide [71,74]. A drug with both antioxidant and anti-inflammatory activities is thus expected to effectively mitigate neuronal degeneration in PD. Minocycline and celastrol both exhibit this salient combination of dual properties. In accordance with their comparable profiles of action, similar effects on DN number and brain dopamine levels were observed with these two drugs in our study. Both drugs were able to halt the loss of DNs and replenish dopamine content to near normal levels in DJ-1A RNAi flies. Minocycline and celastrol were previously shown to protect against MPTP-induced neurotoxicity in mice PD models [75,76], although divergent results exist [77]. Drug studies in toxin-induced models of a disease, however, always bear a confounding effect of the drug reacting directly with the toxin. Thus, a seemingly neuroprotective effect of a drug might be conferred by chemical reaction of the drug with the toxin that results in attenuated toxicity. Here we show in an independent genetic model of PD that the neuroprotective potential of the two drugs is not restricted to pharmacologically induced models. To further increase the likelihood of the drugs' efficacy in idiopathic cases, it might be interesting to test these two drugs in other PD models, which may operate through different pathogenic mechanisms.

Our results suggest that both minocycline and celastrol may prove effective in preventing and/or delaying the progression of PD. However, comparable results were achieved at very different doses, indicating different potency of the two drugs. To produce the same neuroprotective effect as celastrol, minocycline needs to be applied at a more than 10,000-fold higher concentration. This information is relevant especially when the development of protective or preventive medicine is concerned. After all, an augmentation of the drug concentration required to reach the desired therapeutic effect is generally accompanied by an increase of unwanted side effects. Neuroprotective therapy requires the long-term administration of the drug to symptomatic but not yet end-stage patients. In the MPTP mouse model of PD, an oral minocycline concentration of 120 mg/kg day was required to produce a protective effect [75]. The high dose of minocycline given to animals, which is significantly more than what is typically given to humans, is a potential problem. The dose we found to be effective in the fly (50 mg/ml) is also relatively high. Lower doses were tried in our dosing studies but were found to be ineffective (data not shown).

Minocycline has been used for many years as an antibiotic and is considered relatively safe. However, the recommended dose of minocycline is 100 mg to 200 mg/day [78]. High doses of minocycline antibiotics display the same side effects of tetracycline antibiotics. Furthermore, minocycline has a tendency to accumulate in various tissues [79]. Thus, a high dose regimen over longer periods of time might not be recommended. Studies using minocycline should therefore be cautiously evaluated before being used as a basis for human trials. Minocycline has already been incorporated into a clinical investigation by the NINDS/NIH, in which it is administered in the dose of 100 mg in combination with 5g creatine (twice daily) to otherwise untreated PD patients. It remains to be seen the neuroprotective effect of minocycline in a drug-combination regimen. Our results do suggest that a derivative drug with similar antioxidant and anti-inflammatory effects as minocycline but fewer side effects might deserve serious consideration as a PD therapeutic.

Celastrol shows more promising potential as a candidate to be further tested in preclinical PD models. Celastrol has been described previously as having potent antioxidant and anti-inflammatory properties in models of rheumatoid arthritis (RA), Crohn's disease, and Alzheimer's disease (AD) [33,80,81]. Here it is shown that the administration of celastrol in low nanomolar doses provided excellent neuroprotection in a fly model of PD. The effects observed with celastrol were similar to those obtained with minocycline. However, celastrol must be considered superior to minocycline with regard to tolerance in humans. Celastrol has a centuries-long history of use in traditional Chinese medicine. In the present study, celastrol exhibited approximately 10,000-fold higher neuroprotective potency than minocycline. At nanomolar doses (7 μg/kgxd), celastrol was shown to improve learning and memory in a mouse AD model. Higher doses (3 mg/kgxd) were administered to mice, e.g. the MPTP model of PD, without any observable side effects [33,76,80]. Celastrol thus represents a potentially neuroprotective treatment for PD, as it targets at least two main pathogenic factors. It could be administered as single treatment early in the course of the disease and later in combination with L-DOPA or dopamine agonists, where it might be effective at reduced doses. Since celastrol is readily available and comparatively inexpensive, its use may also bear economical benefits by delaying the administration of the more costly symptomatic medications.

### The biogenic amine coQ_10 _fails to show effect in the DJ-1A RNAi model of PD

The reduction in complex I in the mitochondrial electron transport chain in PD patients prompted the therapeutic use of coQ10, the electron acceptor for mitochondrial complex I. CoQ10 itself also has antioxidant properties. Administration of coQ10 to the MPTP mouse model (200 mg/kgxd) and monkey model (15-22 mg/kgxd) attenuated the neurotoxic effects of low dose MPTP [43,46]. Different from these previous studies, our present results do not suggest obvious neuroprotective effects of coQ10 in the DJ-1A RNAi model in the assays we performed. There are several possible explanations for its lack of efficacy in the fly model: 1) Due to its high molecular weight and hydrophobicity, bioavailability of coQ10 is low in humans [82,84], which might also be the case in the fly. CoQ10 might not be stable in yeast paste or could be metabolized rapidly before diffusing into brain tissue. 2) The administered dose of 100 mg/ml might not have been enough. 3) CoQ10 may be inefficacious in the DJ-1A RNAi model of PD simply due to its unique mechanism of action. Previous animal tests of coQ10 were done in MPTP models of PD. MPTP acts through inhibition of complex I of the mitochondrial respiratory chain, and coQ10 is the very electron acceptor of complex I. Based on these, one could assume that the therapeutic efficacy of coQ10 might be restricted to the MPTP or other models with electron transport deficits. However, electron transport dysfunction might not be a primary effect of DJ-1 pathogenesis.

CoQ10 has already been implemented into clinical trials on humans, but the results are inconclusive so far [85,90]. These studies differ significantly in their chosen outcome measures. Some of them are in patients with early disease not yet requiring medication, others in patients with advanced disease already requiring medication, which might not be suitable for neuroprotective trials. Based on our findings, one could extrapolate that if coQ10 did confer neuroprotective effects in humans, it would be limited to specific patient populations.

### NBQX has differential effects on DN number and dopamine levels in *DJ-1A RNAi *flies

After it was realized that excitotoxicity conferred by excess glutamate in the basal ganglia circuit contributes to neuronal degeneration in PD [52], AMPA-selective glutamate receptor (GluR) antagonists have been pursued as potential neuroprotective agents. The use of the AMPA receptor antagonist NBQX in the fly DJ-1A RNAi model yielded divergent results with regards to DN preservation and the attenuation of neurotransmitter depletion. NBQX administration conferred excellent protection against DJ-1A RNAi-induced DN degeneration. However, it had little effect on dopamine level reduction. This divergence might be explained, if dopamine depletion occurs before neuronal degeneration. NBQX might have successfully protected against secondary glutamate-induced excitotoxicity and the subsequent DN cell death, but not reduction of dopamine levels, for which other mechanisms than excitotoxicity might be responsible. It is not clear how DJ-1A dysfunction affects brain dopamine levels. The dopamine biosynthetic enzyme TH clearly is still present in DNs of DJ-1A RNAi animals, since it is detected by immunostaining, although its level appears to be reduced in these neurons. Its activity could also be reduced in DJ-1A mutant condition. These findings thus could be consistent with a hypothetical course of events, in which dopamine depletion precedes DN death. Our results also suggest that excitotoxicity does make certain contribution to neuronal death in PD, but it is clearly not the primary cause. They also argue that simply blocking DN degeneration may not always lead to accompanying restoration of brain DA level and dopaminergic function.

Interestingly, NBXQ treatment (30 mg/ml) appears to be toxic for *Ddc-Gal4/+ *control flies. Reduced neuronal number and decreased dopamine levels were observed. Furthermore, it was noted that NBQX-fed wild type flies exhibited pre-mature mortality and markedly reduced life span (data not shown). In contrast, NBQX-fed DJ-1A RNAi flies did not display any life span reduction, nor did the flies of either genotype treated with any of the other drugs used in this study. It is not clear how NBQX confers toxicity to wild type flies but not the DJ-1A RNAi flies. It is possible that the toxicity is conferred by its competitive mechanism of action. In the control flies with normal glutamate concentrations, AMPA inhibition might reduce the intrinsic activity of glutamatergic neurotransmission to an extent that is incompatible with life, leading to neuronal death by lack of physiological stimulation. In the DJ-1A RNAi fly with presumed excess excitatory glutamatergic activity, competitive inhibition might lead to near normal level of glutamate action. Despite NBQX's receptor selectivity and potency in animal models, it has to be eliminated as a drug candidate for human use because of its poor pharmacokinetics. Its low water solubility at physiological pH, combined with a fast renal excretion, cause crystallization in the kidney at therapeutic doses. The fast excretion also prevents any prolonged systemic activity in humans following oral administration [50]. Therefore newer compounds with better pharmacokinetic properties are being actively pursued [91,93].

### Implications for PD therapy

**Figure 8:**
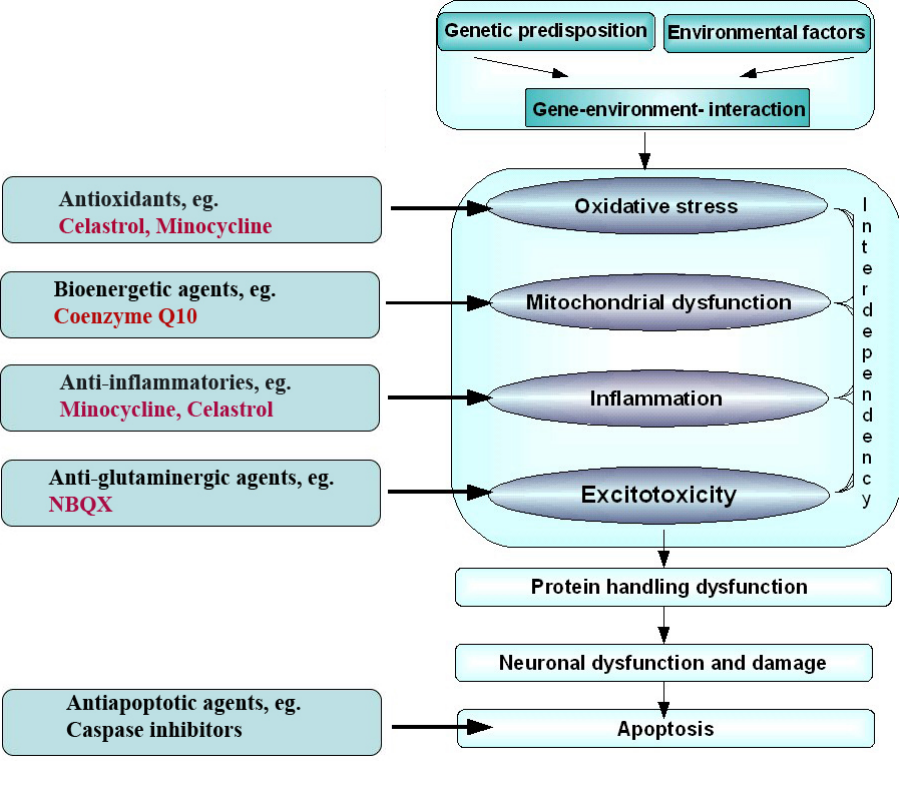
**A diagram depicting possible pathogenic events in PD**. A hypothetical series of molecular events likely contributing to PD pathogenesis is diagrammed. Potential sites of action by the neuroprotective agents used in this study are indicated.

This study presents an *in vivo *system for testing candidate neuroprotective drugs for PD. None of the drugs discussed above would provide a cure for PD. Some of them, however, appear to be able to delay or decelerate disease progression by directly influencing established pathogenic factors. The ability to directly interfere with disease progression would constitute a major improvement to current symptomatic treatments of PD. Preventive/protective medication would be more efficacious when applied early. In the DJ-1A fly model, treatment was offered as early as the beginning of adulthood (day 1). Of the four drugs tested in this study, celastrol may bear the greatest neuroprotective potential for use in idiopathic PD, taking into account its neuroprotective effect and its side-effect profile. The findings of this study appear to favour the hypothesis that oxidative stress may represent an early pathogenic event underlying PD. Oxidative stress is thought to arise from the metabolism of dopamine itself, which produces DOPA-quinones through auto-oxidative processes and leads to the accumulation of neuromelanin and iron, eventually causing mitochondrial dysfunction [66]. All other players theorized to precipitate PD, like protein aggregation, defects in the ubiquitin proteasome system, inflammation, and excitotoxicity, can easily be considered as consequences of oxidative damage. In turn, a cycle of degenerative events ensues through the generation of secondary oxidative stress (Fig. 8). Of the secondary disease-propagating factors, inflammation may have greater and faster destructive potential. Logically, suppression of this prime executor of injury and death, in addition to the primary insult, namely oxidative stress, would confer an effective delay of the neurodegenerative progress. The present results with minocycline and celastrol in the fly DJ-1A model strongly corroborate this hypothesis.

### *Drosophila*: a valuable model for pre-clinical pharmacological studies

The findings of this study support the suitability of *Drosophila *for pre-clinical pharmacological studies. *Drosophila *possesses a complex nervous system of approximately 300,000 neurons, composed of numerous specialized subtypes that utilize major classes of neurotransmitters, receptors, ion channels and signaling pathways found in humans. The complexity of the *Drosophila *brain enables learning and memory and other higher cognitive functions. Moreover, *Drosophila *exhibits numerous sophisticated behaviours, whose impairment can be related to neural pathologies in the disease models [94]. All of these qualities could be employed to qualify and quantify the effects of neuroactive drugs in *Drosophila*. Because of its short lifespan and high reproduction rate, *Drosophila *would be valuable for first-step drug screens. Only those drugs that show promise in *Drosophila *might be moved on to mammals, resulting in faster and more economical animal testing.

To date, a number of studies have been carried out in which flies respond to drugs added to their food. Drug feeding to flies has been described for reserpine and several biogenic amines, such as octopamine, L-DOPA, dopamine, tyramine, tyrosine, 3, 5-diiodotyrosine [95,97]. In the *Drosophila *α-Synuclein model of PD, oral administration of the Hsp90 inhibitor Geldanamycin was shown to have efficacy [59]. Earlier studies in *Drosophila *Huntington's disease models showed that feeding of histone deacetylase inhibitors (either alone or in combination with congo red and cysteine) arrested progressive neuronal degeneration [98,99]. Volatile drugs such as cocaine, alcohol, and nicotine have been administered in aerosols to *Drosophila *and elicited biological responses [100,103].

Our present study further supports *Drosophila *as a useful tool for pre-clinical drug testing for neurodegenerative diseases. Staining of DMC DNs and direct dopamine measurement represent easily measurable and reliable methods for drug screenings in *Drosophila*. In addition, the administration of drug preparations in yeast paste was accepted by the fly and validated as a feasible approach. Ingestion of drugs could be easily monitored, since most drugs bear chromophores that can be detected in the digestive system through the fly's semi-transparent cuticle and also in their faeces. Clearly, experimental procedures could be improved and refined. For example, dose finding in the present study was based on previous empirical values. It might be helpful in the future to measure the effective concentrations of at least a certain number of drug prototypes within the fly's brain tissue after oral administration. This will generate conversion factors, which will facilitate dose estimation in future studies

## Conclusion

*Drosophila *genetic models of PD offer great potential for elucidating disease mechanisms because of their amenability to systematic genetic analysis, including genetic screens for novel players in the disease process. Our results presented here support that *Drosophila *genetic models of PD are also excellent tools for testing the therapeutic potential of drugs. Our analysis showed that minocycline and celastrol, two compounds with antioxidant and anti-inflammatory properties, are effective in attenuating the loss of DNs and reduction of brain dopamine levels in a *Drosophila *DJ-1A model of PD. This result emphasizes oxidative stress and inflammation in DJ-1 pathogenesis. Further testing of minocycline and celastrol in other fly PD models will test the generality of the involvement of oxidative stress and inflammation in inducing the neuropathology observed in these disease models. Second generation compounds based on modification of minocycline or celastrol could offer effective drugs for PD intervention. High throughput compound screening with the fly PD models could also facilitate the discovery of novel PD therapeutics.

## Methods

### *Drosophila *Genetics and Molecular Biology

Adult *Drosophila *of the genotypes *Ddc-Gal4>DJ-1A RNAi, Ddc-Gal4>DJ-1A RNAi + hDJ-1*, and *Ddc-Gal4/+ *were used in most experiments. The *Ddc-Gal4 *line and the *w*^- ^wild type stock were obtained from the Bloomington *Drosophila *Stock Center. Genomic DNA/cDNA constructs were generated to create *UAS-DJ-1A RNAi *transgenics, as described earlier [104]. Analysis of the *UAS-DJ-1A RNAi *and *UAS-hDJ-1 *transgenes was performed as described [20,58].

### Animals and Drug Feeding

Drugs, dissolved in 10% DSMO, were mixed into wet yeast paste. Drug-containing yeast paste was offered together with standard fly food in standard vials housing approximately 10 flies each. All drug preparations were mixed freshly every 2-3 days. Flies were administered celastrol (1 μg/ml, 5 μg/ml, and 20 μg/ml), minocycline (25 mg/ml, 50 mg/ml), NBQX (30 mg/ml), coenzyme Q10 (100 mg/ml), or yeast paste containing 10% DMSO alone as non-treatment control. Drug feeding was carried out at 25°C for 25 days from day 1 after eclosion. Approximately 21-24 flies for each drug and genotype were processed for HPLC-based dopamine measurement after 10 days of treatment. 10-25 fly heads per group were evaluated by immunohistochemistry after 25 days of treatment.

Therapeutic doses were based on dose-finding studies, which compared known oral doses in mammals based on weight, metabolic rate etc. and through parallels to substances previously administered to the fly [95,97,100,103].

### Histology and Immunohistochemistry

For counting DN number with the peroxidase/DAB method, the heads of 1-day and 25-day old adult flies were dissected, formaldehyde-fixed and paraffin embedded as previously described [58]. Serial 10 μm frontal sections were cut from anterior to posterior to include the entire brain. Anti-TH polyclonal antibody (1:100, Pel Freez Biologicals) served as primary antibody. Incubation was carried out over night at 4°C. For subsequent processing, the Vectastain Universal Elite ABC Kit (Vector Laboratories) was used. Immunopositive cells of the dorsomedial cluster at the level of the giant interneuron commissure were counted throughout all sections, using a light microscope (Nikon Eclipse 3000, Japan). Similar methods have been used previously to analyze dopaminergic degeneration in various *Drosophila *models of PD [20,57,59]. For counting DN number with whole-mount confocal microscopy of TH immunostaining, the procedure was carried out essentially as described [64].

### HPLC Measurement of Dopamine

For sample preparation, 10-day-old female fly heads were dissected out under mild CO_2 _anaesthesia, and quickly homogenized in chilled 0.1 M perchloric acid using a motorized, hand-held tissue homogenizer. 3 heads were used per 50 μl perchloric acid sample. Homogenates were frozen immediately on dry ice and stored at -80 degrees prior to HPLC measurement. 21-24 fly heads were measured per genotype and per experiment.

For measurement of dopamine, a modified HPLC protocol for catecholamine measurement was used as described earlier [76,105]. The chilled homogenates of fly heads were centrifuged and 10 μl of supernatant fluid was eluted through an 80 × 4.6 mm C18 column (ESA Inc, Chelmsford, MA, USA). The mobile phase contained 75 mM of NaH_2_PO_4_, 1.5 mM Octanesulfonic acid (OSA), 5% Acetonitrile (pH 3.0). For coulometric electrochemical detection, a two-channel Coulochem II electrochemical detector (ESA, Inc) was used. The flow rate was 1 ml/min. Concentrations of DA were expressed as nanograms per milliliter (ng/ml).

### Climbing Activity and Oxidative Stress Assays

To measure climbing activity, we transferred into empty plastic vials 12 to 20 female flies that have been treated or mock-treated with 20 μg/ml celastrol for 20 days and counted the number of flies climbing up to the top quarter of the vial within a 7-second period, after being bumped to the bottom of vertically standing vials, as previously described [64]. We repeated each measurement as indicated using three independent fly cohorts per genotype. For oxidative stress treatment, cohorts of female flies (n = 20-30 each) treated or mock-treated with 20 μg/ml celastrol for 20 days after eclosion were transferred to plastic vials containing a tissue paper socked with 0.5% H_2_O_2 _prepared in Schneider's insect medium. At least three independent vials were set up for each genotype and drug treatment condition. The survival rate was measured as previously described [20].

### Data Analysis

All data were analyzed using Microsoft Excel and Graph Pad InStat3 software.

The effects of minocycline, celastrol, NBQX and coQ10 ingestion on neuronal survival within the DMC of sectioned or whole-mount fly brains were analyzed with a one-way analysis of variance (ANOVA). Multiple pair-wise comparisons of the means were performed using Dunnett's Multiple Comparison Tests: The effects of the respective drugs on dopamine concentration within the fly head were also analyzed with a one-way analysis of variance (ANOVA) and the Dunnett's Multiple Comparison as post-test. Student's *t*-test was used when a single pair of data was compared. Differences for all data were considered statistically significant at *P *< 0.05. For both studies, mean numbers of surviving neurons or dopamine content, respectively, were plotted as columns. Error bars indicate the standard deviation of the mean (SD). Number of asterisk indicates statistical significance. ***, *P *< 0.0001; ** *P *< 0.01; * *P *< 0.05; no asterisk, *P *> 0.05.

## Authors' contributions

KF carried out the drug studies, the immunohistochemical analysis, and drafted the manuscript. SG participated in whole-mount TH confocal immunofluorescence study and data analysis. YF participated in the drug studies and the immunohistochemical analysis. LY performed the DA measurements. MFB provided the drugs and HPLC analytical methods. BL conceived the study, participated in its design, coordination and execution, and manuscript writing. All authors read and approved the final manuscript.
